# Successful removal of the largest reported intrathoracic lipoma with bilateral extension: a case report

**DOI:** 10.1186/s13019-022-01954-z

**Published:** 2022-08-20

**Authors:** S. M. Tajdit Rahman, Md. Faizul Islam, S. M. Zakirullah Rasha, Abdur Rahim, Tania Binte Ghani Elora, A. K. M. Razzaque

**Affiliations:** 1 Department of Thoracic Surgery, National Institute of Diseases of the Chest and Hospital, Mohakhali, Dhaka, 1212 Bangladesh; 2 General Thoracic and Upper GI Surgery Centre, Green Life Hospital, Dhaka, Bangladesh; 3 Department of Cardiac Surgery, National Institute of Cardiovascular Diseases, Dhaka, Bangladesh

**Keywords:** Lipoma, Intrathoracic lipoma, Giant, Bilateral, Median sternotomy, Case report

## Abstract

**Background:**

Unlike subcutaneous lipomas, thoracic cavity lipomas are extremely rare and can develop to be quite large without causing any symptoms. However, managing massive lipoma that involves both chest cavities is usually challenging, especially when considering the approach for excision.

**Case:**

We report our experience of surgical management of a case of a 46-year-old male with huge intrathoracic lipoma that extends bilaterally and is known to be the largest of such kind. The tumor was resected successfully using median sternotomy. Histological analysis confirmed features of lipoma.

**Conclusion:**

To remove a bilateral intrathoracic lipoma, various surgical approaches have been documented. In our experience, a median sternotomy allows better exposure, which aids in complete surgical extirpation resulting in the prevention of recurrence.

## Introduction

Lipomas are the most prevalent benign mesenchymal tumors, while intrathoracic lipoma is a rare entity. On the other hand, giant lipoma involving both hemithorax is far more unusual, with only a few occurrences documented in the literature [1–3]. Lipomas in the intrathoracic region often go unnoticed, especially if they are small. Despite the fact that a giant lipoma in the chest cavity might cause symptoms, it is frequently identified during a routine medical evaluation [2, 4].

Surgical removal is still the primary line treatment for intrathoracic tumors with a good outcome [1, 2]. However, managing massive lipoma that involves both chest cavities is usually challenging, when considering the approach.

Here, we present our experience of surgical management of a case of a 46-year-old male with huge intrathoracic lipoma that extends bilaterally, displacing the lung upwards. To our knowledge, this is the largest reported intrathoracic lipoma with the bilateral extension, which was removed successfully through standard median sternotomy.

## Case presentation

A 46-year-old hypertensive man presented to our outpatient clinic with a dry cough that had been bothering him for 3 weeks, as well as periodic shortness of breath with chest heaviness when exerted. He has no prior history of chest pain, hemoptysis, or weight loss, nor does he have any relevant medical history. His blood pressure is controlled with medication. He was a cook, non-alcoholic, non-smoker, and having a body weight of 65 kg for his 166 cm height.

Physical examination of the patient revealed absent breath sounds along with dullness on percussion over both the middle and lower chest. Routine blood workup was within the standard limit except for mild anemia and raised ESR.

**Fig. 1:**
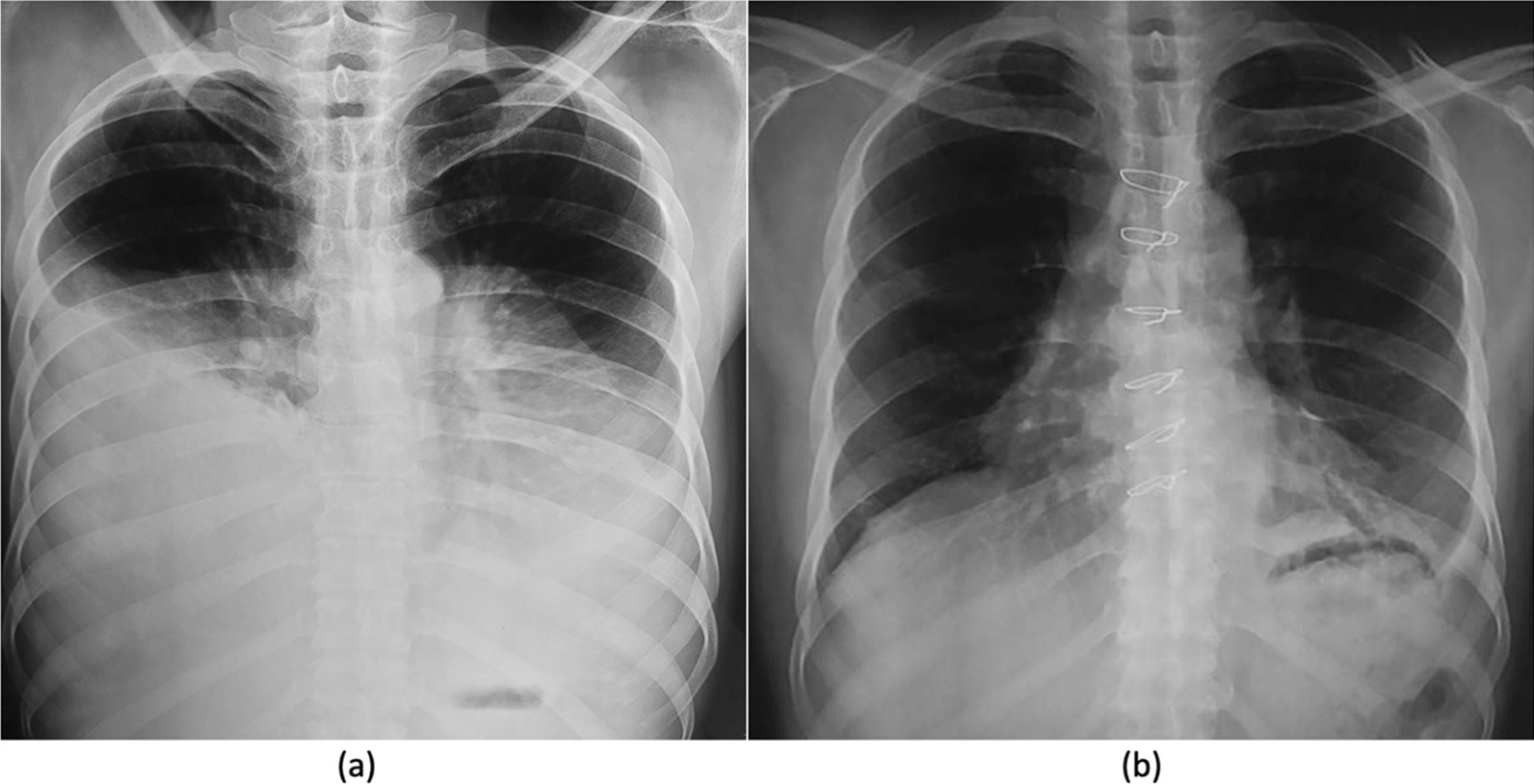
**a** Preoperative chest X-ray shows huge mass occupying both hemithorax and displacing both lungs upwards, **b** postoperative chest X-ray shows no residual mass and well expanded lung after 1 month

**Fig. 2:**
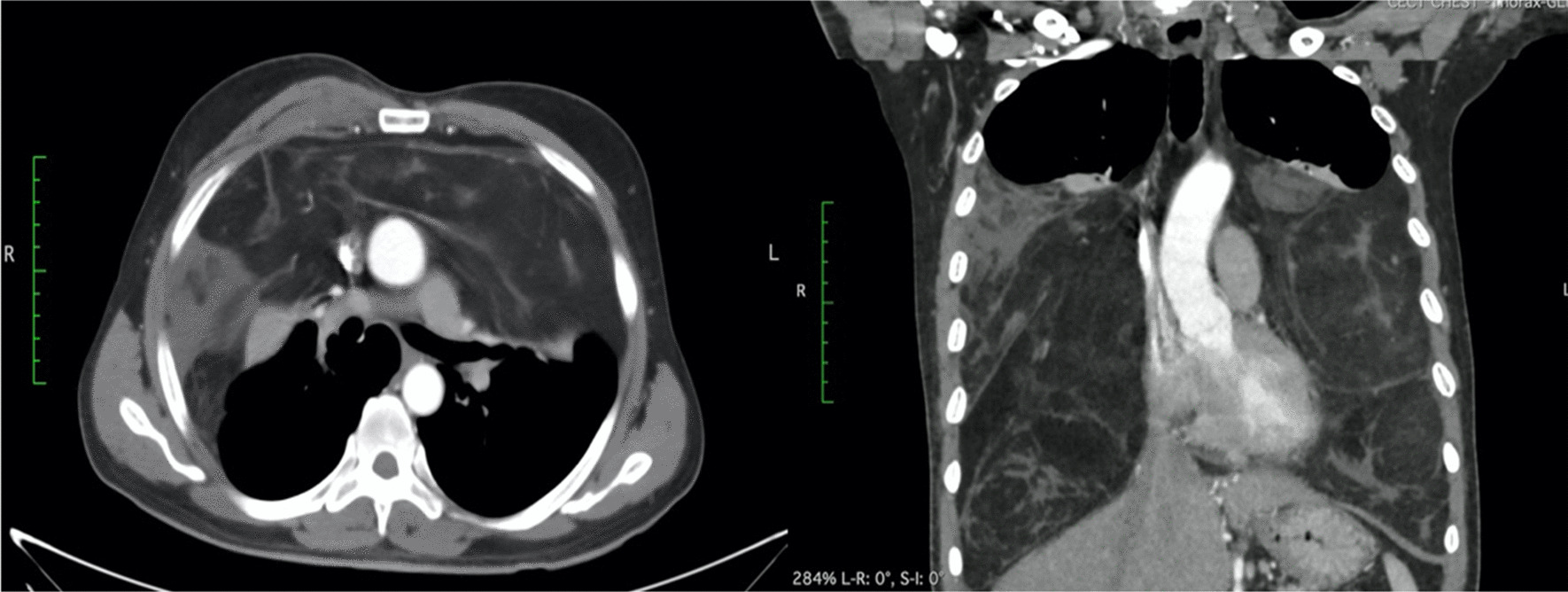
Contrast CT of the chest shows well-defined fat density mass occupying both hemithorax and displaced both lungs upwards

A routine chest X-ray revealed a massive homogenous mass that occupied the lower two-thirds of both chests (Fig. 1a). CECT revealed a substantial fat density area (24 cm transverse, 16 cm cranio-caudal, 17 cm AP dimension) at the mid and lower parts of both chests, involving the mediastinal fat plane compressing and shifting both lungs upwards (Fig. 2). A CT-guided FNAC was performed, and the smear showed polymorph, lymphocytes, and fatty tissue fragments on a blood background, indicating lipoma. Spirometry revealed an obstructive pattern with a forced expiratory volume in first second (FEV_1_) of 1.2 L (42% predicted), forced vital capacity (FVC) 1.7 L (49% predicted) and FEV_1_/FVC ratio of 70%. Preoperative echocardiography showed no evidence of cardiac compression with an ejection fraction of 61%.

**Fig. 3:**
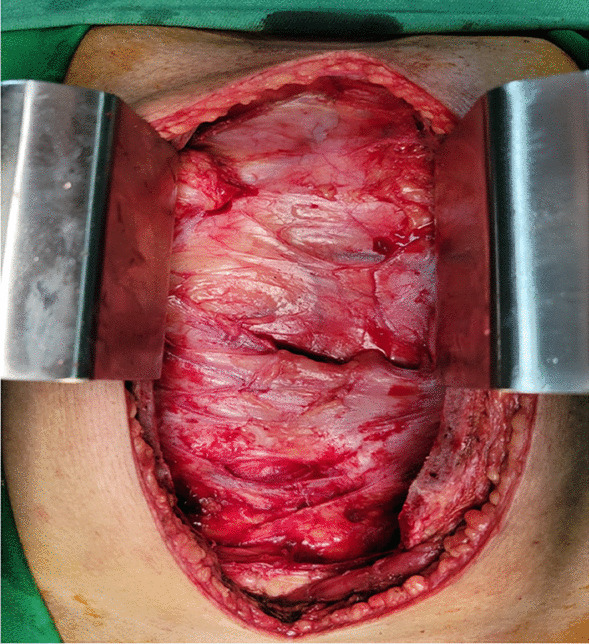
Operative view of the tumor just after median sternotomy

The chest cavity was explored through a standard median sternotomy to provide complete access to both the pleural cavities and the mediastinum in order to remove the massive intrathoracic lipoma. The gross appearance was a thinly encapsulated, lobulated, dull mass with a pale yellow color that had a smooth surface and was soft to the touch (Fig. 3). Mass was continuous with the fat plane of the anterior mediastinum and supra cardiac blood vessel. The right-sided part of the mass was much larger than the left side. There were few adhesions with both lungs, and the lungs were compressed by the tumor. By blunt and sharp dissection and manipulation, tumor from both chest cavity and mediastinum were completely separated and removed. We did not find any obvious vascular pedicle for the tumor during mobilisation except few tiny vessels from mediastinum.

**Fig. 4:**
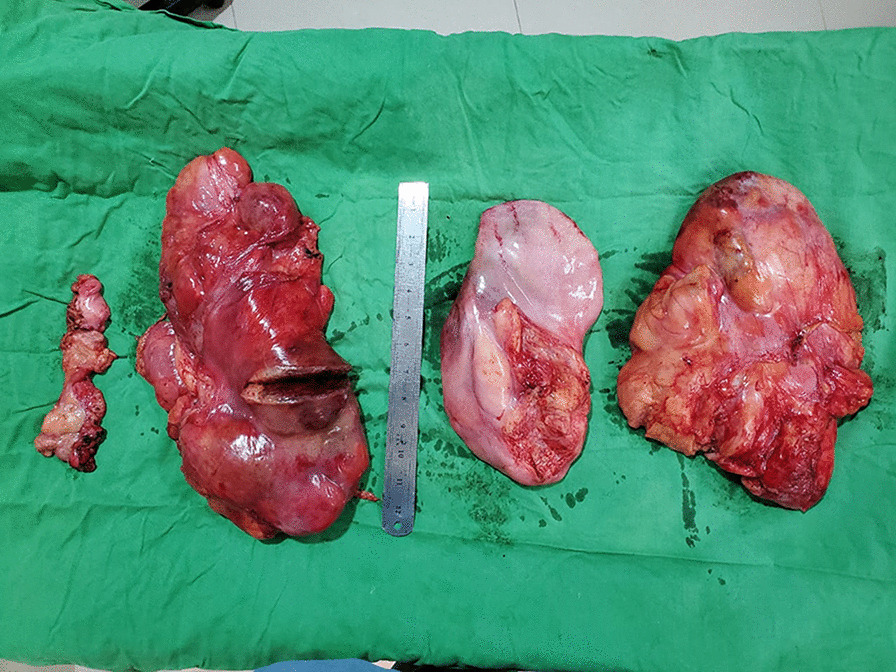
The resected specimen shows three large parts of the tumor. Tumors left to the measuring scale were extracted from left side, whereas tumors right to the scale were removed from right chest cavity

**Fig. 5:**
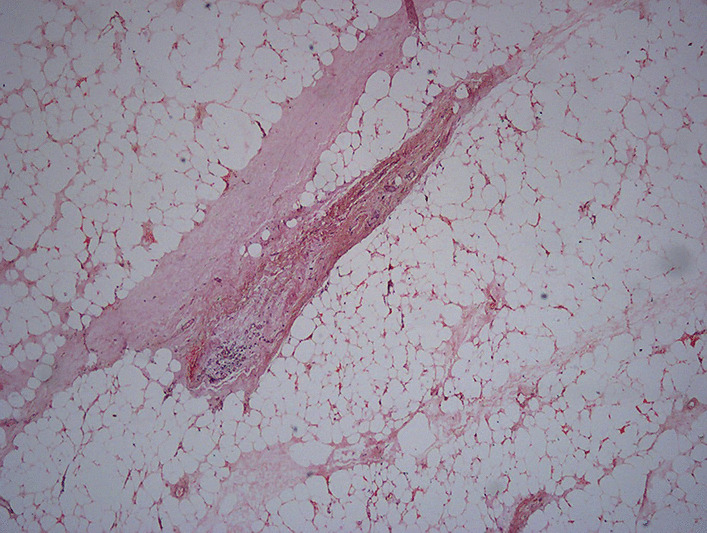
Microscopic examination reveals abundant mature adipose tissue mixed with fibrous tissue

The resected tumor weighed around 5400 g. It contains four lobulated parts, the largest of which is approximately 24*10*8 cm in size. The cut section comprises yellow lobulated fatty tissue (Fig. 4). Final histologic analysis of the tumor revealed an encapsulated tumor with abundant mature adipose tissue, a few scattered strands of fibroconnective tissue,  and multiple foci of fat necrosis (Fig. 5). There was no evidence of abnormal mitosis or invasive cancer.

The postoperative period went smoothly, and on the sixth postoperative day, the patient was discharged home after removing the chest and mediastinal drain with a normal chest X-ray. His physical capacity and respiratory health both improved considerably after he was discharged. After 1 month of removal of the mass, we performed a spirometry to see the improvement of lung function. We observed FVC improved to 2.6 L form 1.7 L and FEV_1_ improved to 2.0 L from preoperative 1.2 L.

At 6 months of follow-up, there was no evidence of recurrence, and the CXR was normal (Fig. 1b).

## Discussion

Lipomas are benign tumors of adipose tissue and the most common benign tumor in adults, accounting for 20% of all benign soft-tissue tumors. Lipoma is primarily made up of immature fat cells, although it can also contain mesodermal components other than adipocytes, such as varying quantities of fibrous tissues and blood vessels [5]. lipomas within the thoracic cavity are extremely uncommon, which was first described by Fothergil [6]. It can be found in mediastinal, diaphragmatic, bronchial, and pulmonary levels [7].

Most people with intrathoracic lipomas are asymptomatic; nevertheless, because lipomas can grow to enormous sizes, they can cause pressure effects, which vary according to the location and size of the lipomas. Symptoms such as dyspnoea and dysphagia might be caused by local compression on neighboring structures such as the trachea or esophagus [7, 8]. Certain authors have even suggested that the mediastinal structure can be compressed by giant intrathoracic lipoma [9, 10]. They can also cause various problems like chest pain and fever, as well as invading intercostal spaces and causing rib lysis [11, 12]. In our case, the patient’s shortness of breath was due to the compressed lung by the massive size of the lipoma, resulting from reduced lung volume. We did not find any intrathoracic lipoma in the literature more than 3500 gm and as large as the reported case by us [1, 13].

Although intrathoracic lipoma is usually detected incidentally in a chest X-ray, a homogeneous fat attenuation mass (50–150 HU) that creates obtuse angles with the chest wall and displaces neighboring pulmonary parenchyma and arteries on a chest CT permits a conclusive diagnosis [1, 2, 5, 11]. Additionally, magnetic resonance imaging (MRI), particularly with fat saturation, is supportive in determining the lipomatous nature of the tumor. Furthermore, MRI helps to distinguish between lipomas and well-differentiated liposarcomas based on margins, signal homogeneity, and septa or nodules [14, 15]. Preoperative percutaneous biopsy and FNAC may not always show the presence of malignant cells due to inadequate sample and cannot completely able to exclude the presence of sarcoma.

When possible, total en-bloc excision of lipoma is the definitive treatment choice for preventing future recurrences. Complete surgical excision using the lateral thoracotomy or standard median sternotomy is the most applied surgical approach for intrathoracic lipoma [16]. Clamshell thoracotomy has also been employed to remove the bilateral intrathoracic lipoma [17]. Whereas, median sternotomy allows complete assessment of both chest cavities and the mediastinum, which ensures en-bloc removal of the encapsulated tumor [13]. In our experience, lateral thoracotomy may affect the exposure in case of a large tumor, resulting in rupture of the capsule [2]. We believe exposure is the main key to ensure en-bloc resection and choosing surgical exposure. In our previous case we did piecemeal excision of the mass through lateral thoracotomy which caused prolong intrathoracic drain. Though clamshell thoracotomy was mentioned in different articles for bilateral intrathoracic tumor, we avoided it due to our concern of upper limit of the tumor and previous disastrous postoperative experiences related to this particular approach. In the literature, video-assisted thoracoscopic surgery (VATS) has been suggested as a technique for thoracic tumors that are usually pedunculated, tiny in size, and do not have infiltrating growth [5].

Intraoperative problems are not infrequent because of severe adhesions with important structures caused by the mass's chronic existence in the thoracic cavity, as in our instance, where it was firmly connected to the innominate vein and superior venacva. Local recurrence of intrathoracic or mediastinal lipomas after resection is infrequent. They may, however, recur locally, and the risk of recurrence following an excision has been documented in the literature to be less than 5% [18]. Despite the fact that intrathoracic lipomas are histologically benign, close monitoring and follow-up are required to identify any recurrence.

## Conclusion

Intrathoracic lipomas are rare benign tumor in contrast of subcutaneous lipoma which is very common. They typically grow very slowly over years without any symptoms and signs. They often found in routine investigations incidentally when they become large. Whereas CT and MRI are usually diagnostic. Although pleural lipoma never evolves towards liposarcoma, surgical resection is still necessary and must be resected completely to prevent further recurrence.

## Data Availability

Not applicable.

## Abbreviations

CXR: Chest X-ray
CECT: Contrast enhanced computed tomography
CT: Computed tomography
FNAC: Fine needle aspiration cytology
FVC: Forced vital capacity
FEV_1_: Forced expiratory volume in first second
MRI: Magnetic resonance imaging
VATS: Video-assisted thoracoscopic surgery

## References

[1] Aldahmashi M, Elmadawy A, Mahdy M (2019). The largest reported intrathoracic lipoma: a case report and current perspectives review. J Cardiothorac Surg.

[2] Rahman SMT, Rahim A, Kibria AA (2020). Unusual cause of large intrathoracic mass in a young male of Bangladesh: a case report of giant intrathoracic lipoma and literature review. Int J Surg Case Rep.

[3] Beshay M, Schmid RA (2004). Intrathoracic giant lipoma. Ann Thorac Surg.

[4] Politis J, Funahashi A, Gehlsen JA, DeCock D, Stengel BF, Choi H (1979). Intrathoracic lipomas. Report of three cases and review of the literature with emphasis on endobronchial lipoma. J Thorac Cardiovasc Surg.

[5] Kamata S, Ishida I, Suzuki Y, Yamada T, Yaegashi H, Oura H (2018). Intrathoracic fibrolipoma resected using complete thoracoscopic surgery: a case report. J Cardiothorac Surg.

[6] Fothergill J (1994). Lipoma of the external thoracic wall. Eur Respir J.

[7] Chen (2015). Intrathoracic giant pleural lipoma: case report and review of the literature. J Cardiothorac Surg.

[8] Cutilli T, Schietoma M, Marcelli VA, Ascani G, Corbacelli A (1999). Giant cervico-mediastinal lipoma. A clinical case. Minerva Stomatol.

[9] Botianu PV, Cerghizan AM, Botianu AM (2015). Giant right intrathoracic myxoid fusocellular lipoma. Case Rep Pulmonol.

[10] Jack AI, Blohm ME, Lye M (1995). An intrathoracic lipoma impairing left ventricular function. Br Heart J.

[11] Andreu C, Yat-Wah P, Fraga J, Olivera MJ, Caballero P (2008). Necrotic lipoma of the posterior mediastinum. Arch Bronconeumol.

[12] Buxton RC, Tan CS, Khane NM, Cuasay NS, Shor MJ, Spigos DG (1998). Atypical transmural thoracic lipoma–CT diagnosis. J Comput Assist Tomogr.

[13] Chen C, Chen M, Liu W, Yuan Y, Yu F (2018). Successful removal of giant mediastinal lipoma and liposarcoma involving both chest cavities. Medicine.

[14] Brisson M, Kashima T, Delaney D, Tirabosco R, Clarke A, Cro S (2013). MRI characteristics of lipoma and atypical lipomatous tumor/well-differentiated liposarcoma: retrospective comparison with histology and MDM2 gene amplification. Skelet Radiol.

[15] Weiss SW, Goldblum JR, Weiss SW, Goldblum JR (2001). Benign lipomatous tumors and liposarcoma. Enzinger and Weiss’s soft tissue tumors.

[16] Marulli G, Rea F, Feltracco P (2007). Successful resection of a giant primary liposarcoma of the posterior mediastinum. J Thorac Oncol.

[17] Hagmaier RM, Nelson GA, Daniels LJ (2008). Successful removal of a giant intrathoracic lipoma: a case report and review of the literature. Cases J.

[18] Sakurai H, Kaji M, Yamazaki K, K. (2008). Suemasu Intrathoracic lipomas: their clinicopathological behaviors are not as straightforward as expected. Ann Thorac Surg.

